# Supplementary material for: Resolving the full spectrum of human genome variation using Linked-Reads
Source: Genome Res. 2019 Apr;29(4):635–45. doi: 10.1101/gr.234443.118 (PMC6442396; doi:10.1101/gr.234443.118)
Supplement: Supplemental Material [file supp_gr.234443.118_Supplemental_longranger-2.2.zip › longranger-2.2.tar.bz2!/lr2.2-public/lib/go/src/code.google.com/vitess/third_party/py/mock-1.0.1.tar.gz!/mock-1.0.1/html/sentinel.html]

Sentinel — Mock 1.0.1 documentation


### Navigation

- index
- next |
- previous |
- Mock 1.0.1 documentation »

# Sentinel¶

sentinel¶
:   The sentinel object provides a convenient way of providing unique
    objects for your tests.

    Attributes are created on demand when you access them by name. Accessing
    the same attribute will always return the same object. The objects
    returned have a sensible repr so that test failure messages are readable.

DEFAULT¶
:   The DEFAULT object is a pre-created sentinel (actually
    sentinel.DEFAULT). It can be used by side\_effect
    functions to indicate that the normal return value should be used.

## Sentinel Example¶

Sometimes when testing you need to test that a specific object is passed as an
argument to another method, or returned. It can be common to create named
sentinel objects to test this. sentinel provides a convenient way of
creating and testing the identity of objects like this.

In this example we monkey patch method to return
sentinel.some\_object:

```
>>> real = ProductionClass()
>>> real.method = Mock(name="method")
>>> real.method.return_value = sentinel.some_object
>>> result = real.method()
>>> assert result is sentinel.some_object
>>> sentinel.some_object
sentinel.some_object
```

### Table Of Contents

- Sentinel
  - Sentinel Example

#### Previous topic

Helpers

#### Next topic

Mocking Magic Methods

### This Page

- Show Source

### Quick search


Enter search terms or a module, class or function name.

### Navigation

- index
- next |
- previous |
- Mock 1.0.1 documentation »

© Copyright 2007-2012, Michael Foord & the mock team.
Last updated on Nov 05, 2012.
Created using Sphinx 1.1.3.
